# Supplementary material for: Antimicrobial Properties of Lepidium sativum L. Facilitated Silver Nanoparticles
Source: Pharmaceutics. 2021 Aug 27;13(9):1352. doi: 10.3390/pharmaceutics13091352 (PMC8466285; doi:10.3390/pharmaceutics13091352)
Supplement: Supplementary file 1 [file pharmaceutics-13-01352-s001.zip › pharmaceutics-1340365-supplementary.pdf]

pharmaceutics

Supplementary materials: Antimicrobial Properties of Lepidium sativum L. Facilitated Silver Nanoparticles

Samir Haj Bloukh, Zehra Edis , Hamid Abu Sara and Mustafa Ameen Alhamaidah

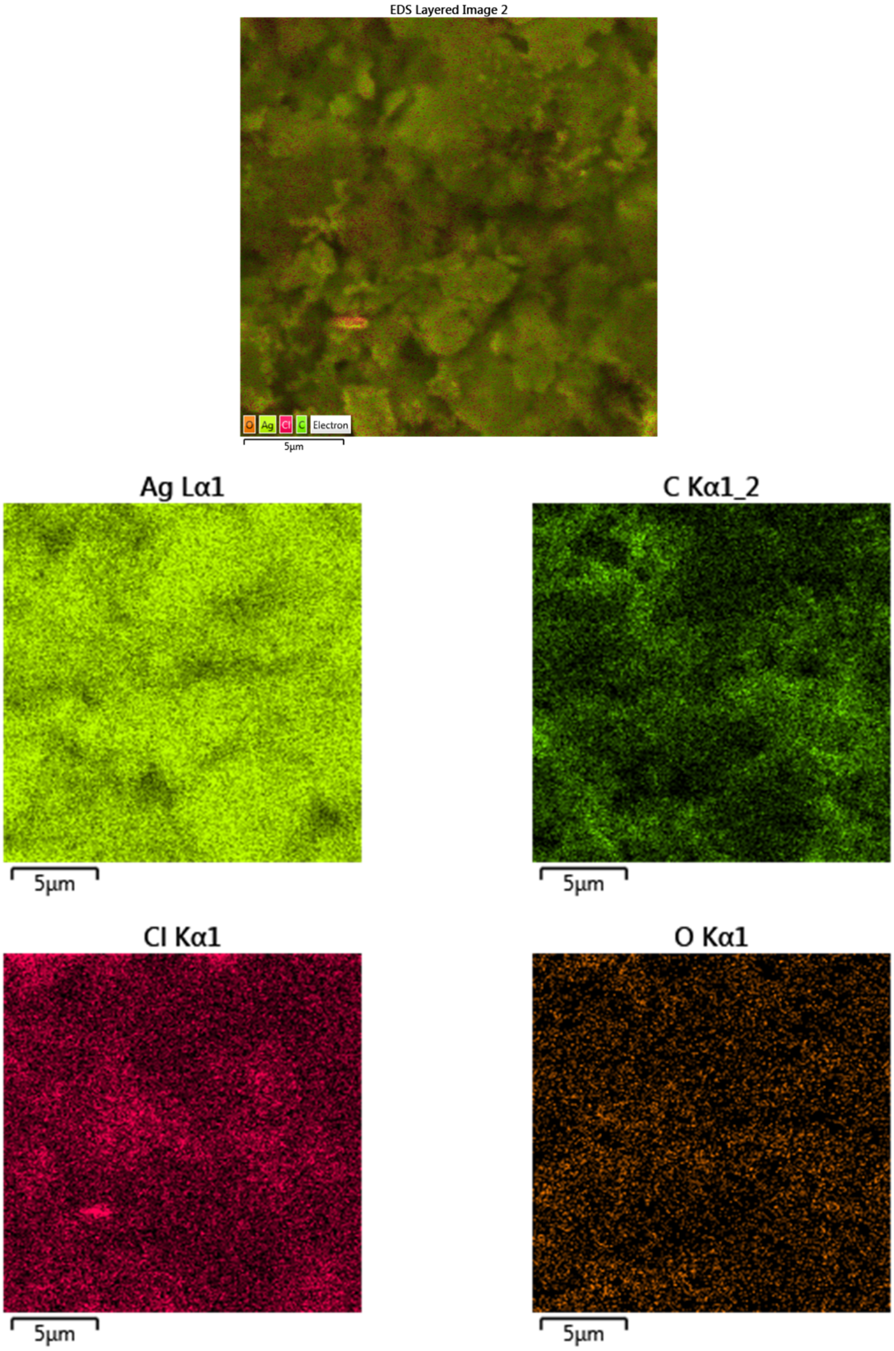

Figure S1: Layered EDS.

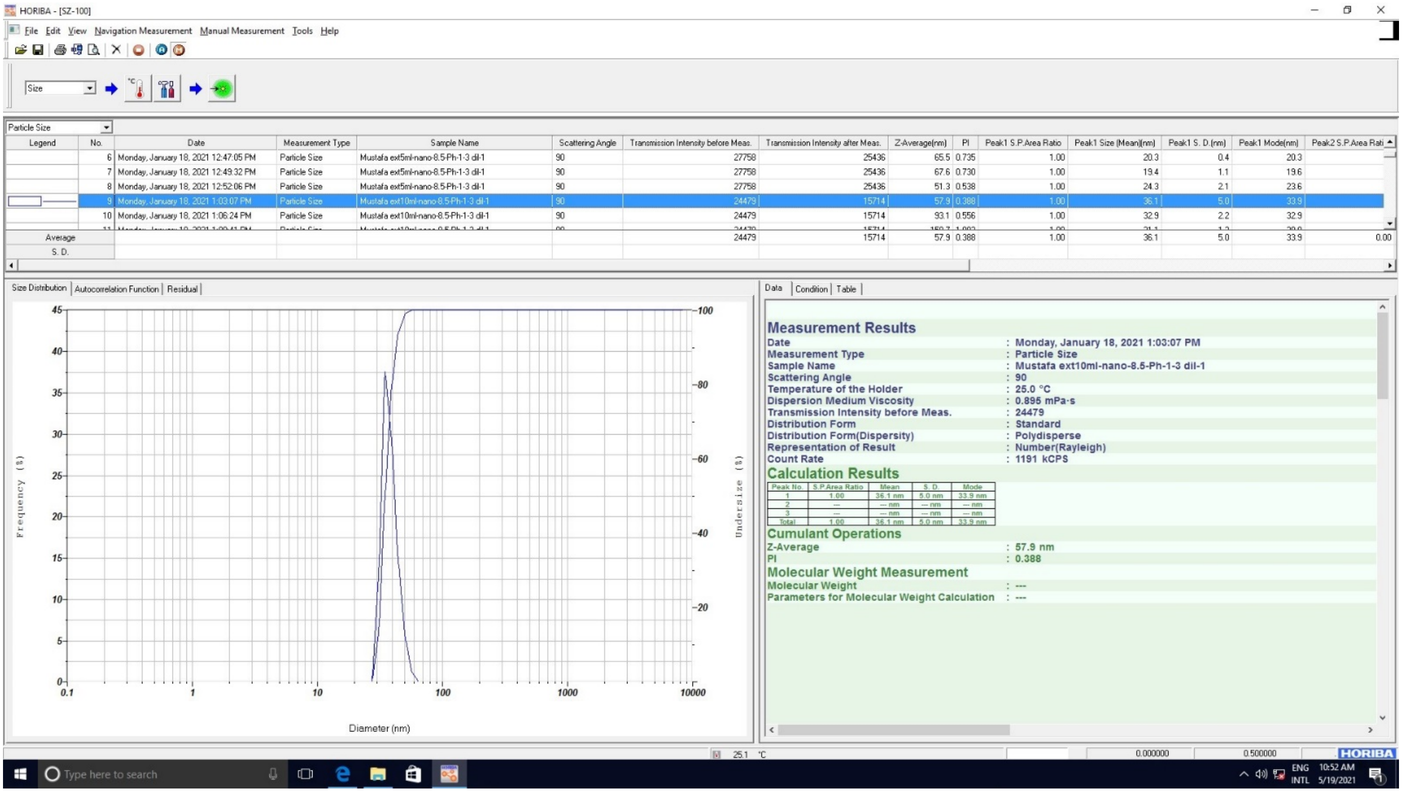

Figure S2: DLS.

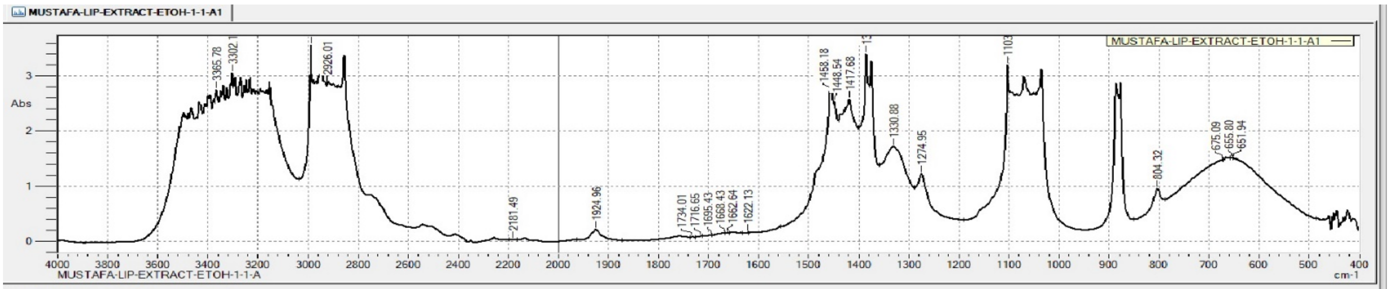

Peak Pick Table | Point Pick Table | Pause Record

No.	Peak	Intensity	Corr. Intensity	Base (H)	Base (L)	Area	Corr. Area	Area color	Comment
1	651.94	1.507	0.015	653.87	486.06	159.526	-1.268	Auto	
2	655.80	1.509	0.003	657.73	653.87	5.817	0.005	Auto	
3	675.09	1.481	0.012	788.89	673.16	132.579	2.598	Auto	
4	804.32	0.969	0.296	850.61	788.89	38.784	2.919	Auto	
5	1103.28	3.203	0.576	1190.08	1099.43	79.868	-61.457	Auto	
6	1274.95	1.234	0.334	1288.45	1190.08	62.777	-4.674	Auto	
7	1330.88	1.712	0.486	1359.82	1288.45	102.595	17.913	Auto	
8	1384.89	3.403	0.787	1400.32	1379.10	53.968	2.167	Auto	
9	1417.68	2.577	0.312	1427.32	1400.32	62.437	2.531	Auto	
10	1448.54	2.535	0.093	1450.47	1438.90	27.065	-0.021	Auto	
11	1458.18	2.720	0.052	1556.55	1456.26	87.590	-61.727	Auto	
12	1622.13	0.156	0.001	1631.78	1620.21	1.772	0.003	Auto	
13	1662.64	0.156	0.002	1666.50	1658.78	1.191	0.007	Auto	
14	1668.43	0.154	0.004	1693.50	1666.50	3.608	0.072	Auto	
15	1695.43	0.111	0.003	1712.79	1693.50	1.940	0.026	Auto	
16	1716.65	0.092	0.006	1726.29	1712.79	1.132	0.024	Auto	
17	1734.01	0.079	0.005	1737.86	1726.29	0.881	0.020	Auto	
18	1924.96	0.214	0.192	1963.53	1872.88	6.812	4.885	Auto	
19	2181.49	0.029	0.004	2196.92	2164.13	0.884	0.056	Auto	
20	2926.01	2.857	0.010	2927.94	2924.09	11.002	0.020	Auto	
21	2989.66	3.561	0.856	3037.89	2983.88	92.509	-16.259	Auto	
22	3302.13	3.065	0.231	3305.99	3298.28	22.490	0.626	Auto	
23	3365.78	2.751	0.156	3373.50	3361.93	30.908	1.079	Auto	

Figure S3: FTIR-Pure LS extract in ethanol.

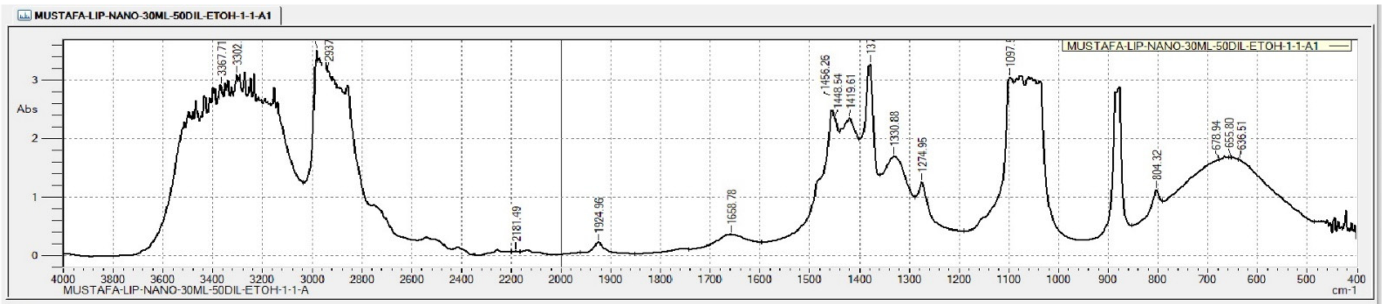

Peak Pick Table | Point Pick Table | Pause Record

No.	Peak	Intensity	Corr. Intensity	Base (H)	Base (L)	Area	Corr. Area	Area color	Comment
1	636.51	1.634	0.015	638.44	486.06	164.353	-4.162	Auto	
2	655.80	1.681	0.007	657.73	653.87	6.473	0.013	Auto	
3	678.94	1.637	0.012	790.81	677.01	148.544	2.238	Auto	
4	804.32	1.123	0.282	852.54	790.81	46.787	2.255	Auto	
5	1097.50	3.048	0.242	1190.08	1091.71	103.409	-62.508	Auto	
6	1274.95	1.250	0.334	1288.45	1190.08	64.878	-4.622	Auto	
7	1330.88	1.696	0.472	1359.82	1288.45	102.177	17.412	Auto	
8	1379.10	3.279	1.606	1400.32	1359.82	91.234	22.868	Auto	
9	1419.61	2.347	0.296	1438.90	1400.32	84.727	5.632	Auto	
10	1448.54	2.343	0.044	1450.47	1438.90	25.623	-0.084	Auto	
11	1456.26	2.664	0.410	1597.06	1450.47	110.415	-77.123	Auto	
12	1658.78	0.359	0.186	1741.72	1597.06	34.895	11.104	Auto	
13	1924.96	0.224	0.186	1961.61	1851.66	8.742	4.850	Auto	
14	2181.49	0.069	0.004	2198.85	2164.13	2.316	0.072	Auto	
15	2937.59	3.164	0.021	2939.52	2922.16	53.853	0.277	Auto	
16	2980.02	3.507	0.307	3035.96	2976.16	119.884	-17.095	Auto	
17	3302.13	3.091	0.144	3317.56	3298.28	56.538	1.088	Auto	
18	3367.71	2.905	0.164	3373.50	3361.93	32.980	1.254	Auto	

Figure S4: FTIR-LS-AgNP in ethanol.
